# Diagnostic criteria of periprosthetic joint infection: a prospective study protocol to validate the feasibility of the 2018 new definition for Chinese patients

**DOI:** 10.1186/s12891-019-2941-1

**Published:** 2019-11-20

**Authors:** Haitao Guan, Chi Xu, Jun Fu, Ming Ni, Xiang Li, Wei Chai, Libo Hao, Yonggang Zhou, Jiying Chen

**Affiliations:** 1 Department of Orthopaedics, Chinese People’s Liberation Army General Hospital (301 Hospital), 28 Fuxing Rd, 100853 Beijing, People’s Republic of China; 2 Medical School of Nankai University, Tianjin, China

**Keywords:** Periprosthetic joint infection, New definition, Prospective study, Diagnosis, Chinese patients

## Abstract

**Background:**

Periprosthetic joint infection (PJI) is a challenging complication following total joint arthroplasty (TJA), and the diagnostic criteria remains controversial. The 2018 new definition proposed in May 2018 consists of new diagnostic criteria for PJI. We conducted a retrospective study and demonstrated that the new definition could improve the diagnostic efficiency in Chinese patients. However, missing data led to bias in the previous retrospective study. Therefore, this prospective study is designed to further validate the feasibility of 2018 new definition (and its modified version) for Chinese patients.

**Methods/design:**

This is a single-centre, prospective diagnostic study with 1 year of follow-up. The patients enrolled in the trial will be divided into a PJI group and an Aseptic group based on the eligibility criteria. We will recruit at least 70 patients for each group from October 2019 to October 2020. Blood samples, synovial fluid samples and intraoperative variables of all the included patients will be collected to assess various indicators. We will integrate the results of the various tests and examine the diagnostic efficiency (sensitivity and specificity) of five diagnostic criteria.

**Discussion:**

We design the prospective study in the hope of reducing the bias caused by missing data. Therefore, the prospective study will further support the conclusion of our preceding retrospective study. The results of this study will be submitted to a peer-reviewed journal for publication.

**Conclusion:**

Through this prospective study, we will validate the feasibility of the 2018 new PJI definition (and its modified version) for Chinese patients and determine the optimal method of PJI diagnosis.

**Trial registration:**

Chinese Clinical Trial Registry, ChiCTR1900025395. Registered on 25 August 2019.

## Background

With the rapid growth of the ageing population, the number of patients requiring total joint arthroplasty (TJA) for the treatment of osteoarthritis or other degenerative joint diseases has been rapidly increasing [1–4]. Periprosthetic joint infection (PJI) is the most devastating postoperative complication and is harmful to the health and economic status of patients [5–8]. Although strict aseptic techniques and rational antibiotic management after surgery are widely followed in clinical practice, PJI is still common in patients undergoing failed TJA [8]. It has been reported that 20.4% of patients undergo revision total knee arthroplasty (TKA) due to PJI in the United States [9].

The diagnosis of PJI is still controversial, and orthopaedic surgeons are searching for a more accurate diagnostic method that could help in determining whether revision surgery is appropriate for patients. At present, the diagnostic criteria proposed by the Musculoskeletal Infection Society (MSIS) and then partly modified at the International Consensus Meeting (ICM) in 2013 is widely used in the diagnosis of PJI [10–12]. Due to the advances in the knowledge of orthopaedic surgeons regarding PJI and the development of the means of detection, it is necessary to further improve the diagnostic efficiency of the MSIS and ICM criteria. In 2018, researchers proposed a new definition of PJI by establishing an evidence-based and weight-adjusted scoring system [13]. The scoring system involved intraoperative variables, preoperative serum markers and synovial markers. Researchers evaluated the new definition on a total of 422 patients and concluded that the new definition provided better sensitivity and similar specificity compared with the MSIS and ICM criteria [13]. The new definition was reviewed and further altered by the International Consensus on Orthopedic Infections [14].

To validate the feasibility of the new PJI definition for Chinese patients, we retrospectively studied a cohort of 98 patients in a PJI group and 165 patients in an aseptic loosening group [15]. The included patients all underwent revision total knee (TKA) and hip arthroplasty (THA) between January 2015 and August 2017 in our hospital. For patients in both groups, the sensitivity of the new PJI definition reached 94.9%, which was significantly higher than that of the MSIS and ICM criteria. The specificity of the new definition remained at 95.2%. Therefore, we concluded that the 2018 new definition of PJI was also appropriate for use with Chinese patients [15].

However, in the retrospective study, part of the data were lost due to the loss of medical records, and some synovial markers were not available because they are not widely measured in clinical practice. To reduce the bias caused by these missing data and to improve the quality of our research, we designed and planned to conduct a prospective study that could further validate the feasibility of the new PJI definition for Chinese patients.

## Methods

### Study design and objectives

This single-centre trial is designed as a prospective diagnostic study. The objectives of this study are to supplement and improve the results of the preceding prospective study and to validate the feasibility of the 2018 new PJI definition for Chinese patients. The study is nonrandomized, and all patients serve as their own controls. From October 2019 to October 2020, we will recruit patients who agree to participate in the trial in the Department of Orthopaedics of the Chinese PLA General Hospital (301 hospital). Blood samples, synovial fluid samples and intraoperative findings will be collected for patients meeting the inclusion criteria. All of the samples and clinical data will be processed and analysed by a double-blind method. The major criteria of PJI (a sinus tract communicating with the joint or two positive cultures of the same organism) are identical among various diagnostic criteria [10–14, 16]. Therefore, we will use the major criteria as the gold standard to divide the enrolled patients into a PJI group and an Aseptic group. Then, we will compare the diagnostic efficiency of the respective minor criteria among the 2018 new definition (before revision and after revision), the MSIS criteria, the ICM criteria and the IDSA (Infectious Diseases Society) criteria (Tables 1, 2, 3, 4 and 5) [10–14, 16]. All patients included in our study will be followed for at least 1 year.

**Table 1:** New scoring based definition for periprosthetic joint infection (PJI)

Major criteria (at least one of the following)				Decision
Two positive cultures of the same organism				Infected
Sinus tract with evidence of communication to the joint or visualization of the prosthesis				
Preoperative Diagnosis	Minor criteria		Score	Decision
	Serum	Elevated CRP or D-Dimer	2	≥6 Infected 2–5 Possibly Infected^a^ 0–1 Not Infected
		Elevated ESR	1	
	Synovial	Elevated Synovial WBC or LE	3	
		Positive Alpha-defensin	3	
		Elevated Synovial PMN (%)	2	
		Elevated Synovial CRP	1	
Intraoperative Diagnosis	^a^Inconclusive pre-op score or dry tap		Score	Decision
	Preoperative Score		–	≥6 Infected 4–5 Inconclusive^b^ ≤3 Not Infected
	Positive Histology		3	
	Positive Purulence		3	
	Single Positive Culture		2	

Proceed with caution in: Adverse local tissue reaction, crystal deposition disease, slow growing organisms

^a^For patients with inconclusive minor criteria, operative criteria can also be used to fulfill definition for PJI

^b^Consider further molecular diagnostics such as next-generation sequencing

**Table 2:** Proposed 2018 ICM criteria for PJI (modified version of the 2018 new definition)

Major criteria (at least one of the following)				Decision
Two positive growth of the same organism using standard culture methods				Infected
Sinus tract with evidence of communication to the joint or visualization of the prosthesis				
Minor Criteria	Threshold		Score	Decision
	Acute^b^	Chronic		Combined preoperative and postoperative score: ≥6 Infected 3–5 Inconclusive^a^ <3 Not Infected
Elevated CRP (mg/L)	100	10	2	
or				
D-Dimer (ug/L)	Unknown	860		
Elevated Serum ESR (mm/hr)	No role	30	1	
Elevated Synovial WBC (cells/μL)	10,000	3000	3	
or				
Leukocyte Esterase	++	++		
Or				
Positive Alpha-defensin (signal/cutoff)	1.0	1.0		
Elevated Synovial PMN (%)	90	70	2	
Single Positive Culture			2	
Positive Histology			3	
Positive Intraoperative Purulence^c^			3	

^a^Consider further molecular diagnostics such as Next-Generation Sequencing

^b^These criteria were never validated on acute infections

^c^No role in suspected adverse local issue reaction

**Table 3:** Musculoskeletal Infection Society (MSIS) criteria proposed in 2011

Major criteria	
1. There is a sinus tract communicating with the prosthesis	
2. A pathogen is isolated by culture from at least two separate tissue or fluid samples obtained from the affected prosthetic joint	
Minor criteria	
1. Elevated serum erythrocyte sedimentation rate (ESR) and serum C-reactive protein (CRP) concentration	
2. Elevated synovial leukocyte count	
3. Elevated synovial neutrophil percentage (PMN%)	
4. Presence of purulence in the affected joint	
5. Isolation of a microorganism in one culture of periprosthetic tissue or fluid	
6. Greater than five neutrophils per high-power field in five high-power fields observed from histologic analysis of periprosthetic tissue at× 400 magnification.	

PJI exists when one of the major criteria occurs or four of the six minor criteria occur

**Table 4:** International Consensus Meeting (ICM) criteria proposed in 2013

Major criteria	
1. Two positive periprosthetic cultures with phenotypically identical organisms	
2. A sinus tract communicating with the joint	
Minor Criteria	
1. Elevated serum C-reactive protein (CRP) and erythrocyte sedimentation rate (ESR)	
2. A single positive culture	
3. Elevated synovial fluid white blood cell (WBC) count or ++ change on leukocyte esterase test strip	
4. Elevated synovial fluid polymorphonuclear neutrophil percentage (PMN%)	
5. Positive histological analysis of periprosthetic tissue	

PJI exists when one of the major criteria occurs or three of the five minor criteria occur

**Table 5:** Infectious Diseases Society of America (IDSA) criteria

Definition of PJI	
1. The presence of a sinus tract that communicates with the prosthesis 2. The presence of acute inflammation based on histopathologic examination of periprosthetic tissue at the time of surgical debridement or prosthesis removal. 3. The presence of purulence surrounding the prosthesis. 4. Two or more intraoperative cultures or combination of preoperative aspiration and intraoperative cultures that yield the same organism. Growth of a virulent microorganism (eg, *S. aureus*) in a single specimen of a tissue biopsy or synovial fluid may also represent PJI. 5. The presence of PJI is possible even if the above criteria are not met; the clinician should use his/her clinical judgment to determine if this is the case after reviewing all the available preoperative and intraoperative information	

### Primary outcome

The diagnostic efficiency (sensitivity) of PJI with the minor criteria of the 2018 new definition (before and after revision).

### Secondary outcomes

The following will be assessed:

Specificity, true positive, true negative, false positive, false negative, positive predictive value and negative predictive value of the new definition (before and after revision).

Sensitivity, specificity, true positive, true negative, false positive, false negative, positive predictive value and negative predictive value of the MSIS criteria.

Sensitivity, specificity, true positive, true negative, false positive, false negative, positive predictive value and negative predictive value of the ICM criteria.

Sensitivity, specificity, true positive, true negative, false positive, false negative, positive predictive value and negative predictive value of the IDSA criteria.

The outcomes above are calculated from the data of patients’ medical record information and laboratory tests. We would like to summarize these outcome data as follows. Patients’ basic information such as demographics, comorbidities and surgery information will be collected first through reviewing patients’ medical records or asking patients themselves. Then we will collect data of serum indicators, synovial indicators and intraoperative indicators of each patient. Serum indicators consist of serum C-reactive protein (CRP), serum D-Dimer, serum interleukin-6 (IL-6) and erythrocyte sedimentation rate (ESR). Synovial indicators consist of synovial white blood cell (WBC), leukocyte esterase (LE), synovial alpha-defensin, synovial polymorphonuclear neutrophil percentage (PMN%) and synovial CRP. Intraoperative indicators consist of histology (pathological data from freezing tissue slices), existence of purulence and microorganism culture. These outcome data will be used to determine whether PJI occurs according to various criteria. Finally, we will acquire result of sensitivity, specificity, true positive, true negative, false positive, false negative, positive predictive value and negative predictive value of five diagnostic criteria of PJI. The concrete methods of data acquisition and processing are clearly introduced in the subsequent ‘Recruitment and intervention’ section.

### Inclusion criteria

1. Patients who meet the major criteria of the new definition, that is, patients who have a sinus tract communicating with the joint or two positive cultures of the same organism in tissue or synovial fluid, will be included in the PJI group.
2. Patients who undergo a one-stage revision surgery for aseptic reasons (with no evidence of sinus tracts or two identical positive cultures) will be included in the Aseptic group.

### Exclusion criteria

1. Patients aged<18 years old.
2. Patients whose synovial fluid is not available through preoperative aspiration or intraoperatively.
3. Patients undergoing a failed one-stage revision surgery caused by subsequent PJI.
4. Patients who have a follow-up period of less than 1 year.
5. Patients visiting the hospital with an antibiotic-loaded cement spacer in their joints.
6. Patients with a long preoperative history of antibiotic treatment.
7. Patients who have undergone multiple surgeries on the same joint.
8. Patients with a history of inflammatory joint diseases, such as rheumatoid arthritis and ankylosing spondylitis.
9. Patients with active infection of other parts of the body or serious systemic infection.
10. Patients with malignant tumour or severe disease of the cardiovascular system, pulmonary system or other system.
11. Diabetic patients with poor glucose control.

### Recruitment and intervention

**Fig. 1:**
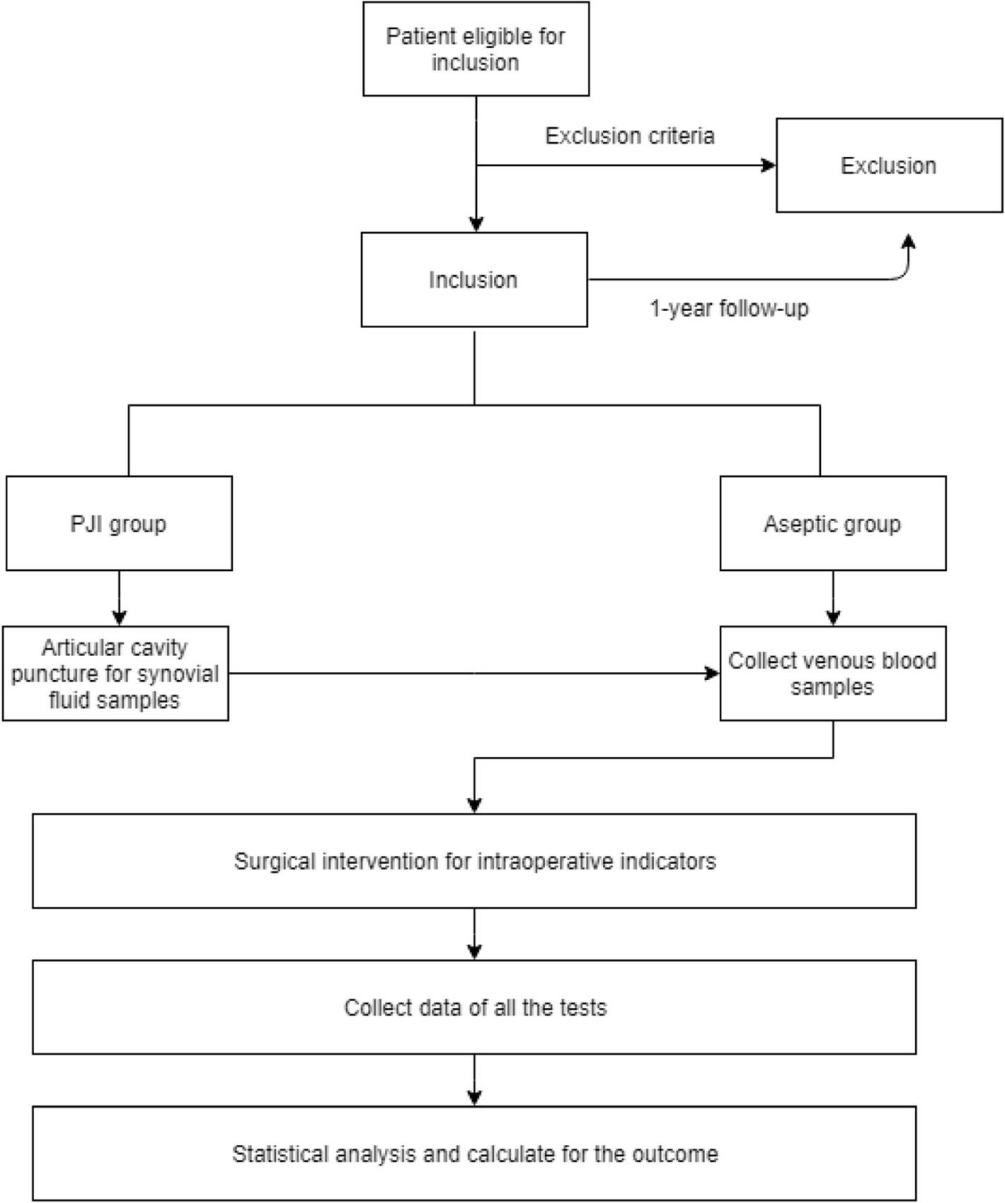
Study flow diagram. The process of recruitment, intervention, follow-ups, data collecting and statistical analysis in our study is presented. PJI = periprosthetic joint infection

The study flow is illustrated in Fig. 1. The patient’s medical history will be evaluated by one orthopaedic surgeon on the day of admission. Patients who meet the eligibility criteria will be asked to sign an informed consent form if they agree to participate in the trial. Then, the patient will be numbered and assigned to the PJI group or Aseptic group based on the inclusion criteria. The recruitment of patients will begin in October 2019 and end in October 2019.

**Fig. 2:**
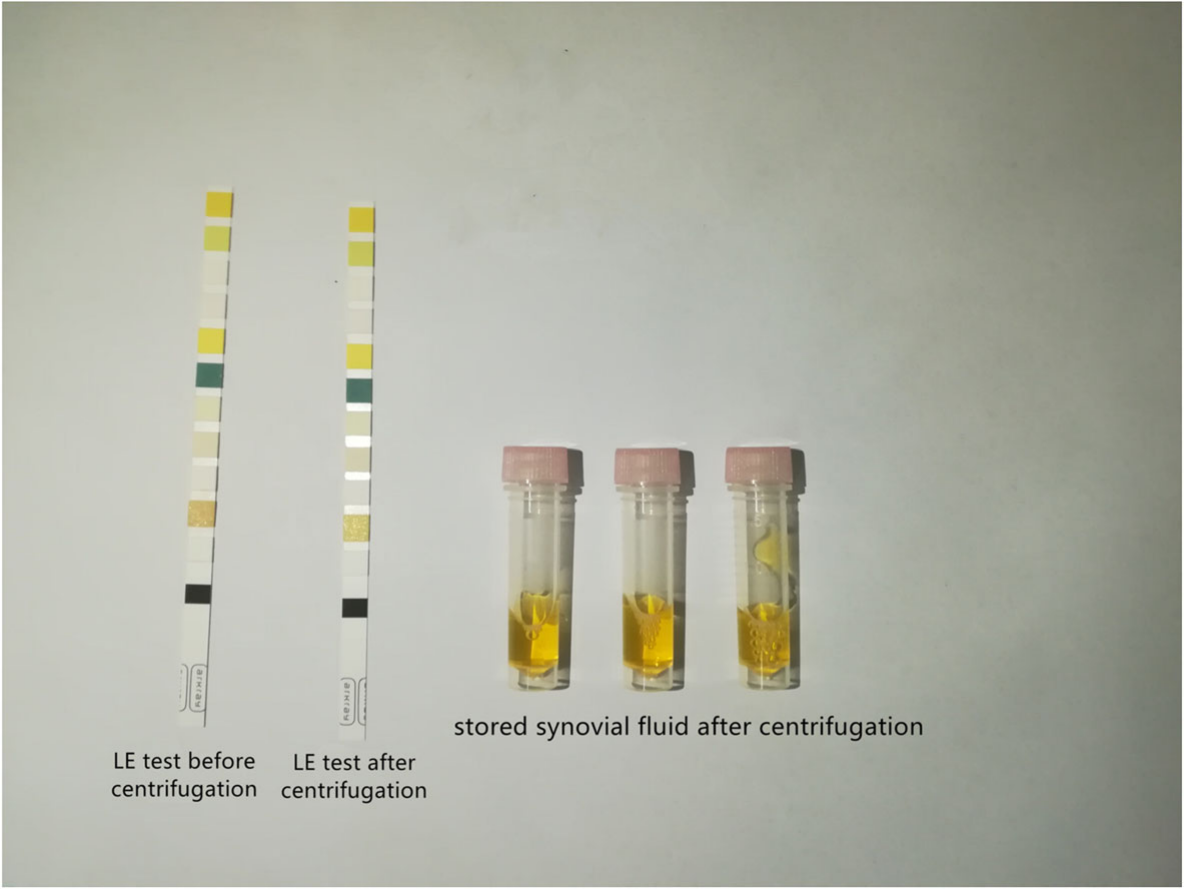
Management of synovial fluid and test results of leukocyte esterase (LE). The synovial fluid samples acquired preoperatively and intraoperatively are all centrifuged and divided into 3–4 samples. The centrifuged samples will be stored at − 80 C freezer for further study. Synovial fluid samples before centrifugation and after centrifugation are separately used for the test of LE. The results of LE test will be photographed and recorded

A venous blood sample and synovial fluid sample will be obtained from the patients before surgery. After admission, a blood sample will be extracted by a nurse and immediately tested by the Clinical Laboratory of our hospital. The serum CRP, serum IL-6, serum D-Dimer levels and ESR will be determined. Generally, synovial fluid samples will be extracted from PJI patients by an experienced resident physician before admission. Portions of the extracted synovial fluid will be sent to the Division of Microbiology for the culture of microorganisms and to the Clinical Laboratory for routine synovial tests. Therefore, we will acquire the culture results, drug sensitivity results, synovial WBC counts and synovial PMN% when patients visit the hospital. The remaining synovial fluid will be centrifuged and stored in a − 80 C freezer for further study [17]. A drop of centrifuged synovial fluid will be obtained to examine LE [18] (Fig. 2). Importantly, we will not preoperatively extract synovial fluid from patients in the Aseptic group to prevent iatrogenic infection. Therefore, we will not take the culture results before surgery into account when patients are included in the Aseptic group. If two positive cultures of an identical organism are found intraoperatively for a patient in the Aseptic group, the patient will be excluded from the Aseptic group and included in the PJI group instead.

During the operation, we will collect three periprosthetic tissue samples from each patient for microorganism culture and three tissue samples for histological analysis. Meanwhile, synovial fluid will be intraoperatively collected and processed as described above. An experienced surgeon will record whether purulence is present in the articular cavity.

The stored synovial fluid of the patients will be numbered, and the details will be recorded by an investigator responsible for managing synovial fluid samples. The number of the synovial fluid sample should be identical to the number of the corresponding patient. Then, the synovial fluid will be delivered to an investigator blinded to the details of the sample who will measure the synovial alpha-defensin and synovial CRP levels by the ELISA (enzyme linked immunosorbent assay) method [19–21].

All of the above data will be delivered to an investigator for statistical analysis. The investigator will only receive the patient number and various indicator values and will determine whether the patient is suffering from PJI based on the test results using different diagnostic criteria. Five kinds of diagnostic criteria are shown in the Tables 1, 2, 3, 4 and 5. The patient meeting one of these criteria will be considered a PJI patient according to this criteria. Using the five diagnostic criteria, the investigator responsible for the statistical analysis will finally determine five diagnoses respectively corresponding to five diagnostic criteria for each patient. Statistical results will be sent to researchers for calculation of the primary and secondary outcomes.

### Blind reading of outcomes

The information of patients in our study will be accessible to the surgeon responsible for numbering and including patients, the investigator managing synovial fluid samples and the investigator collecting the test results. The results of objective indicators, including demographic characteristics, serum CRP, serum IL-6, serum D-Dimer, ESR, culture results (tissue or synovial fluid), drug sensitivity, histological analysis, synovial white blood cell (WBC) counts and synovial polymorphonuclear neutrophil percentage (PMN%), will be directly recorded by the investigator collecting test results.

The remaining indicators will be first processed and evaluated by an investigator blinded to the details of the patients and samples. Changes on the leukocyte esterase (LE) test strips will be evaluated by an investigator without knowledge of patient information. Synovial alpha-defensin and synovial CRP will be measured by an investigator who obtains only the samples and sample numbers. However, the presence of purulence will be determined by the surgeons who are in control of patient information. These processed data will be collected by the investigator who subsequently collects the test results.

The investigator responsible for statistical analysis will also be blinded to patient information. Nevertheless, it is impossible for the surgeons and resident physicians who extract synovial fluid to be blinded.

### Sample size

Sample size calculations were performed using PASS software version 19.0.2 (licensed by NCSS, LLC). In our retrospective study, the sensitivity and specificity of the new definition were 94.9% (95% confidence interval [CI] 87.9–98.1%) and 95.2% (95% CI 90.3–97.7%), respectively [15]. Based on the results of our calculation, we should analyse the data of 60 patients in the PJI group and 58 patients in the Aseptic group. Therefore, we need to include at least 63 patients in the PJI group and 61 patients in the Aseptic group under the assumption of 5% withdrawal and loss to follow-up. In case some patients are excluded from the study for other clinical reasons, we decided to include at least 70 patients in each group.

### Statistical analysis

The statistical analysis will be carried out with IBM SPSS statistics version 20 (SPSS Inc., Armonk, NY). Descriptive statistics will be determined as numbers (frequencies), medians (lower quartile, upper quartile) and means ± standard deviations. Differences in demographic characteristics will be analysed with the Chi-square test or Student’s t-test. Statistical significance will be defined as a *p* value < 0.05. We will calculate the true positive, true negative, false positive, false negative, positive predictive value and negative predictive value of the various criteria and finally obtain the sensitivity and specificity results.

### Data monitoring

All data will be monitored by the Chinese PLA General Hospital, and the accuracy and authenticity of data will be ensured. Participants will be included in our trial based on strict filtering criteria and the signing of an informed consent form.

### Patient and public involvement

The patients and the public are not directly involved in the development of the research question. These populations will also not be involved in the design, recruitment and implementation of the trial.

## Discussion

The misdiagnosis of PJI is harmful to patients’ health and economic status [22–24]. Orthopaedic surgeons have widely explored and summarized the diagnosis of PJI [21, 25–27]. However, an accurate determination can still not be made using the current diagnostic criteria (for example, the MSIS and ICM criteria) [28–30]. The 2018 new definition of PJI and its modified version have provided a weight-adjusted and evidence-based scoring system that can integrate different indicators to evaluate whether PJI has occurred [13, 14]. It is necessary to evaluate the new criteria using different patient populations before its large-scale application in clinical practice.

We retrospectively studied a cohort of 98 patients in a PJI group and 165 patients in an Aseptic group and finally demonstrated a diagnostic sensitivity of 94.9% and specificity of 95.2% for the new criteria [15]. Therefore, we determined that the 2018 new definition of PJI showed improved diagnostic efficiency in Chinese patients. Nevertheless, a partial loss of data in the study led to limitations, and we decided to prospectively evaluate the new definition to improve the reliability of our conclusion. In addition, we will compare the 2018 new definition with its revised version in this prospective study. The methods and expected process of the study have been detailed in this protocol and will be used to ensure the quality of the study.

There are still limitations of the study. Inevitably, surgeons performing surgical interventions and some of the investigators who collect data will not be blinded to patient information. The surgeons may subjectively select the clinical samples, which could lead to biased results of the microbial cultures and histological analysis. Another limitation is the previously mentioned loss of data. It is difficult to preoperatively or intraoperatively extract synovial fluid from some patients, especially from patients in the Aseptic group. These patients will be excluded from our study, and the number of included patients is a concern. We will extend the recruitment time if we encounter a lack of samples.

## Conclusion

The results of this prospective study are expected to strongly support the preceding retrospective study carried out by us. Through the study, we will validate the feasibility of the 2018 new PJI definition (and its modified version) for Chinese patients and determine the optimal method of PJI diagnosis.

## Data Availability

This is a study protocol, and this trial is presently in progress. Thus, no data are currently available.

## Abbreviations

CI: Confidence interval
CRP: C-reactive protein
ELISA: Enzyme linked immunosorbent assay
ESR: Erythrocyte sedimentation rate
ICM: International Consensus Meeting
IDSA: Infectious Diseases Society
IL-6: Interleukin-6
LE: Leukocyte esterase
MSIS: Musculoskeletal Infection Society
PJI: Periprosthetic joint infection
PMN%: Polymorphonuclear neutrophil percentage
THA: Total hip arthroplasty
TJA: Total joint arthroplasty
TKA: Total knee arthroplasty
WBC: White blood cell
